# Distinct Encoding of Reward and Aversion by Peptidergic BNST Inputs to the VTA

**DOI:** 10.3389/fncir.2022.918839

**Published:** 2022-07-04

**Authors:** Marta E. Soden, Joshua X. Yee, Beatriz Cuevas, Ariana Rastani, Jordan Elum, Larry S. Zweifel

**Affiliations:** ^1^Department of Pharmacology, University of Washington, Seattle, WA, United States; ^2^Department of Psychiatry and Behavioral Sciences, University of Washington, Seattle, WA, United States

**Keywords:** ventral tegmental area (VTA), bed nucleus of the stria terminalis (BNST), NTS, CRF, NkB, Tac2, Crh, neuropeptide

## Abstract

Neuropeptides play an important role in modulating mesolimbic system function. However, while synaptic inputs to the ventral tegmental area (VTA) have been extensively mapped, the sources of many neuropeptides are not well resolved. Here, we mapped the anatomical locations of three neuropeptide inputs to the VTA: neurotensin (NTS), corticotrophin releasing factor (CRF), and neurokinin B (NkB). Among numerous labeled inputs we identified the bed nucleus of the stria terminalis (BNST) as a major source of all three peptides, containing similar numbers of NTS, CRF, and NkB VTA projection neurons. Approximately 50% of BNST to VTA inputs co-expressed two or more of the peptides examined. Consistent with this expression pattern, analysis of calcium dynamics in the terminals of these inputs in the VTA revealed both common and distinct patterns of activation during appetitive and aversive conditioning. These data demonstrate additional diversification of the mesolimbic dopamine system through partially overlapping neuropeptidergic inputs.

## Introduction

The mesolimbic dopamine system plays an important role in the regulation of a multitude of motivated behaviors and reinforcement learning (Wise, 2004). The diverse functions of this system are shaped in part by heterogeneity in midbrain cell types, projection targets, and inputs. The VTA is an aggregate of dopamine-, GABA-, and glutamate-releasing neurons (Morales and Margolis, 2017), some of which co-release a combination of these neurotransmitters (Trudeau et al., 2014). There is also cellular diversity within these populations as defined by differential gene expression, projection patterns, and function at the level of behavioral regulation (Poulin et al., 2018, 2020; Heymann et al., 2020; Phillips et al., 2022). Although there are clear differences in the functionality of dopamine-, glutamate-, and GABA-releasing neurons of the VTA, these neurons receive inputs from many common sources (Faget et al., 2016), though the strength of connections of these inputs onto specific cell types, as measured by the amplitude of evoked responses using optogenetic circuit mapping, is variable (Bocklisch et al., 2013; Hjelmstad et al., 2013; Jennings et al., 2013; Nieh et al., 2016; McHenry et al., 2017; Yang et al., 2018; Soden et al., 2020).

In addition to the regulation of the VTA by a variety of neurotransmitter systems, there are multiple neuropeptides that modulate the function of neurons in this region, in particular dopamine-producing neurons (Kalivas, 1985; Borgland et al., 2010; Werkman et al., 2011; Margolis et al., 2014; Tyree and de Lecea, 2017). Dopamine neurons in the VTA express a wide variety of neuropeptide receptors (Chung et al., 2017), some of which define functionally distinct subpopulations of dopamine neurons (Heymann et al., 2020). It has long been appreciated that neuropeptide signaling in the VTA can influence behavior (Kelley and Cador, 1988); however, a comprehensive analysis of the sources for many of these neuropeptides, and whether they are derived from distinct or similar brain regions is lacking.

To begin to address this, we mapped the anatomical locations of three neuropeptidergic inputs to the VTA: NTS, NkB, and CRF. In the VTA, receptors for NTS, NkB, or CRF (*Ntsr1, Tacr3*, and *Crhr1*, respectively) are expressed predominantly on dopamine neurons (Woodworth et al., 2018b; Heymann et al., 2020) and bath application or infusion of agonists for these receptors alters the physiology and firing of dopamine neurons in slice (Jiang et al., 1994; Wanat et al., 2008; Werkman et al., 2011) and dopamine release recorded *in vivo* (Kalivas et al., 1983; Marco et al., 1998; Wanat et al., 2013). These neuropeptides all have stimulatory actions upon receptor binding but have been shown to influence different behaviors (Stoessl et al., 1991; Binder et al., 2001; Dedic et al., 2018; Hupalo et al., 2019) suggesting that they may engage distinct mesolimbic circuits.

To map the sources of NTS, NkB, and CRF to the VTA, we used a cell-type specific retrograde tracing strategy and found that these inputs are derived from numerous unique and overlapping sources. We identified the BNST as the only brain region that provides a significant source of all three neuropeptides, containing a similar number of inputs of all three. Approximately half of the BNST neurons projecting to the VTA expressed a combination of two of the three peptides examined, but only about 2% expressed all three. We observed that the terminals of these inputs to the VTA show dynamic calcium responses during both appetitive and aversive conditioning, including responses to operant actions, cues, and rewards, and the development of responsiveness to a conditioned stimulus following Pavlovian threat conditioning. Consistent with the partially overlapping expression of the neuropeptide encoding genes, these calcium dynamics showed both common and distinct features between the peptide populations.

## Results

### Anatomical Distribution of NTS, NkB, and CRF Inputs to the VTA

**Figure 1:**
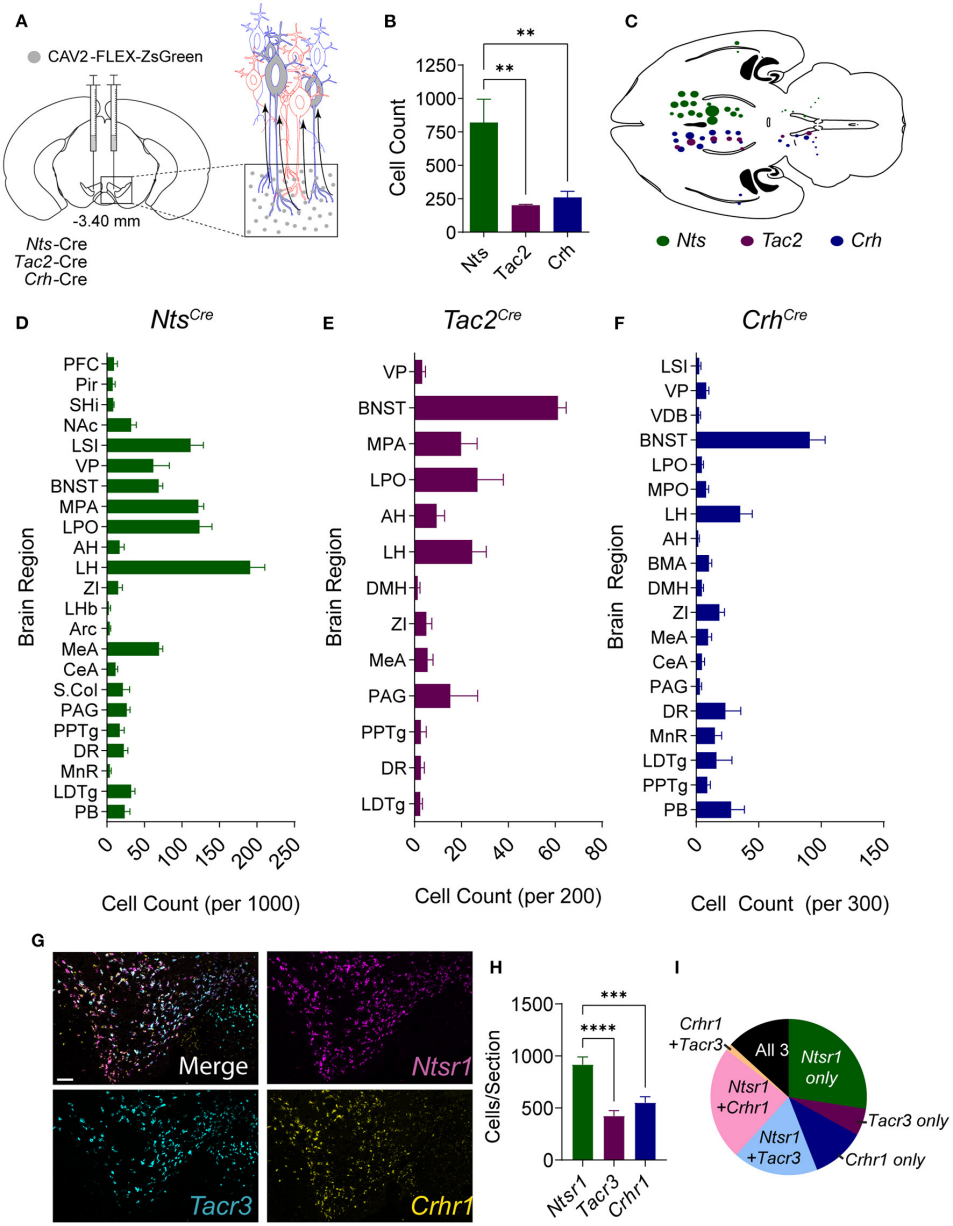
Anatomical distribution of NTS, NkB, and CRF inputs to the VTA. **(A)** Schematic of injection strategy. CAV-FLEX-zsGreen was injected into the VTA of *Nts*-Cre, *Tac2*-Cre, and *Crh*-Cre mice. The virus is taken up by axon terminals and transported retrogradely to the cell body, and zsGreen expression is turned on in Cre-positive neurons. **(B)** Total retrogradely labeled cells throughout the brain [*n* = 5 Nts, 5 Tac2, and 6 Crh mice, One-way ANOVA *F*_(2, 13)_ = 6.141, *P* = 0.0133, Bonferroni multiple comparisons **P < 0.01]. **(C)** Horizontal brain schematic showing distribution of retrogradely labeled cells in each Cre line. The size of the dot is proportional to the number of cells labeled in each region. **(D–F)** Counts of retrogradely labeled cells in each brain region normalized to the total number of labeled cells labeled per mouse. **(G)** Example images of *in situ* hybridization in the VTA for peptide receptors *Ntsr1* (magenta), *Tacr3* (cyan), and *Crhr1* (yellow). Scale bar = 100 μm. **(H)** Number of VTA cells positive for each peptide receptor [*n* = 11 total sections from *N* = 4 mice, One-way ANOVA *F*_(2, 30)_ = 17.03, *P* < 0.0001, Bonferroni multiple comparisons ***P < 0.001, ****P < 0.0001]. **(I)** Pie chart showing proportion of neurons expressing each peptide receptor alone or multiple peptide receptors. Data in bar graphs are presented as mean ± SEM. PFC, prefrontal cortex; Pir, piriform cortex; SHi, septohippocampal nucleus; NAc, nucleus accumbens; LSI, lateral septum intermediate; VP, ventral pallidum; VDB, ventral diagonal band; BNST, bed nucleus of the stria terminalis; MPA, medial preoptic area; LPO, lateral preoptic area; AH, anterior hypothalamic area; LH, lateral hypothalamic area; BMA, basomedial amygdala; DMH, dorsomedial hypothalamus; ZI, zona incerta; LHb, lateral habenula; Arc, arcuate nucleus; MEA, medial amygdala; CeA, central amygdala; S.Col, superior colliculus; PAG, periaquiductal gray; PPTg, pedunculopontine tegmental nucleus; DR, dorsal raphe; MnR, median raphe; LDTg, laterodorsal tegmental nucleus; PB, parabrachial nucleus.

To establish the sources of NTS, NkB, and CRF to the midbrain, we injected mouse lines in which expression of Cre recombinase is under the control of the endogenous neuropeptide-encoding genes for NTS (*Nts-*Cre), NkB (*Tac2*-Cre), or CRF (*Crh*-Cre) with the retrograde transducing virus canine adenovirus serotype 2 (CAV2) containing a conditional expression cassette for the fluorescent protein ZsGreen (CAV2-FLEX-ZsGreen) into the VTA (Figure 1A). Brain-wide analysis of ZsGreen labeled cells revealed broadly distributed inputs for all three neuropeptides (Figures 1B–F; Supplementary Figure 1), with NTS-producing neurons showing the largest number of projection neurons to the VTA (Figure 1B) from the largest number of brain regions (Figures 1C–F).

Given the differences in the total number of inputs from NTS, NkB, and CRF neurons, we asked whether there is a difference in the number of cells in the VTA that express the receptors for these neuropeptides. To address this, we performed fluorescent *in situ* analysis (RNAscope) for the three main receptors for these neuropeptides, *Ntsr1, Tacr3*, and *Crhr1*. Consistent with our input mapping, we found that cells expressing *Ntsr1* were more abundant than those expressing either *Crhr1* or *Tacr3* (Figures 1G–I). Approximately 44% of labeled cells expressed a single peptide receptor, 43% expressed two of the three receptors, and 13% expressed all 3 (Figure 1I).

### The BNST Is a Shared Source of NTS, NkB, and CRF Inputs

**Figure 2:**
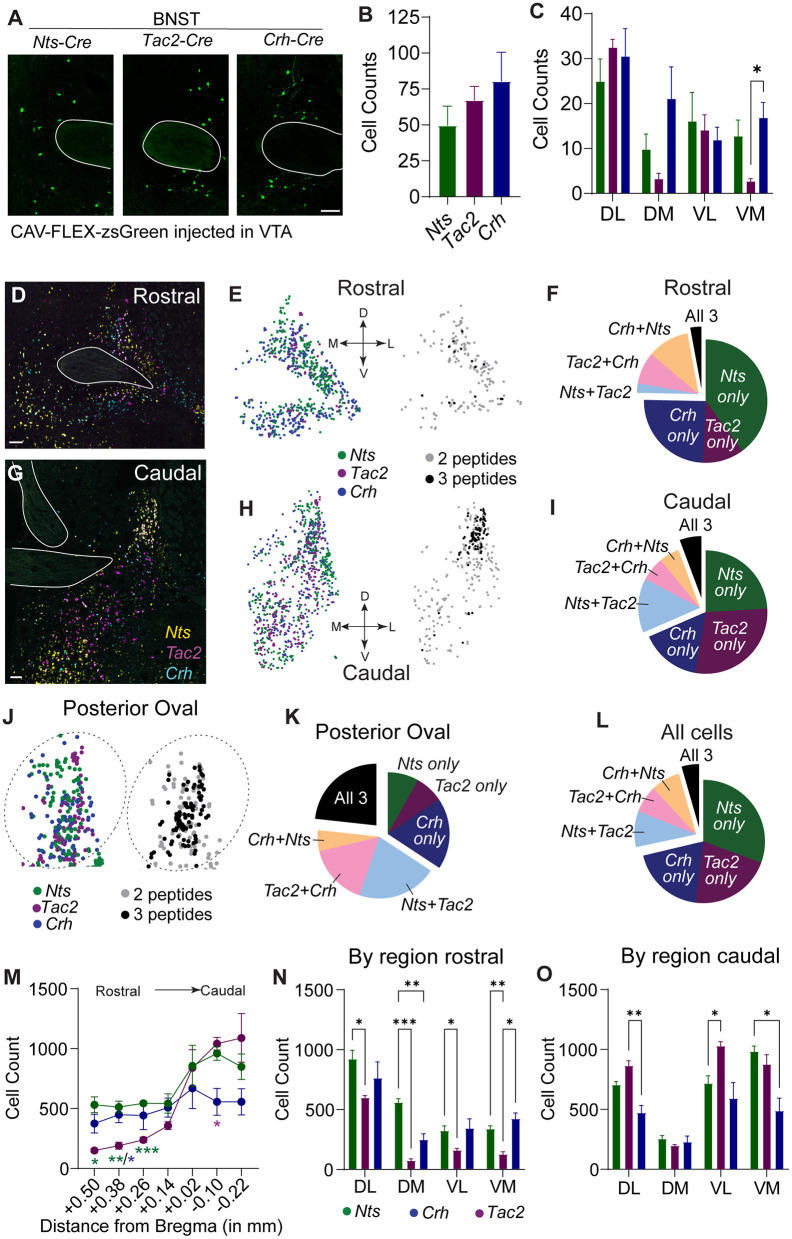
Spatial distribution of *Nts, Tac2*, and *Crh* expression in the BNST. **(A)** Example images showing zsGreen retrogradely labeled *Nts, Tac2*, and *Crh* neurons in the BNST. Scale bar = 100 μm. **(B)** Total number of retrogradely labeled cells in the BNST in each Cre line (*n* = 5 Nts, 5 Tac2, and 6 Crh mice). **(C)** Distribution of retrogradely labeled cells in the dorsolateral, dorsomedial, ventrolateral, and ventromedial BNST [2 way RM ANOVA, effect of Cre line: *F*_(2, 14)_ = 4.181, *P* = 0.0377, effect of region: *F*_(2.292, 32.09)_ = 9.698, *P* = 0.0003, Tukey's multiple comparisons *P < 0.05]. **(D,G)** Example images of *in situ* hybridization for *Nts* (yellow), *Tac2* (magenta), and *Crh* (cyan) in the rostral and caudal BNST. Scale bar = 100 μm. **(E,H,J)** Representation of spatial distribution of peptide-expressing neurons in example images (each dot represents one cell). Left, all cells expressing each peptide. Right, cells expressing 2 or 3 peptides only. Plots in J are zoom of plots in **(H)**. **(F,I,K,L)** Pie charts showing proportion of neurons expressing each peptide alone or multiple peptides in different regions of the BNST (cells counted in *n* = 7 sections from each of 4 mice). **(M)** Rostral-caudal distribution of neurons expressing each peptide [cells counted in *n* = 7 sections from each of 4 mice, 2 way RM ANOVA, Interaction *F*_(12, 54)_ = 3.797, *P* = 0.0003, Tukey's multiple comparisons *P < 0.05, **P < 0.01, ***P < 0.001]. **(N,O)** Number of peptide-expressing neurons in each region of the rostral and caudal BNST [2 way RM ANOVA, rostral: Interaction *F*_(6, 27)_ = 7.330, *P* = 0.0001, caudal: Interaction *F*_(6, 27)_ = 8.940, *P* = 0.0001, Tukey's multiple comparisons *P < 0.05, **P < 0.01, ***P < 0.001]. Data in bar graphs are presented as mean ± SEM.

We observed several shared anatomical sources of NTS, NkB, and CRF to the VTA, but the BNST displayed the largest proportion of these shared inputs where similar numbers of neurons expressing each peptide were labeled (Figures 2A,B). The LH was the largest NTS input, the third largest NkB input, and the second largest CRF input; however, the number of labeled LH NTS cells greatly outnumbered both CRF and NkB producing neurons (Supplementary Figure 2A). Analysis of the location of retrogradely labeled BNST inputs revealed that NTS, NkB, and CRF are broadly distributed throughout the structure with approximately equal distribution across the rostral-caudal axis (Supplementary Figure 2B). Within the subdivisions of the BNST (Supplementary Figure 2C), we observed the largest number of VTA-projecting neurons in the dorsal lateral aspect of the structure in all three Cre lines (Figure 2C), but also found significant differences in the anatomical location of the retrogradely labeled cells between lines, notably in the ventromedial subdivision (Figure 2C).

To determine whether the differences in the distribution of neuropeptide inputs from some anatomical locations in the BNST reflect general differences in the location of these neuropeptide producing neurons, we performed RNAscope *in situ* analysis. Like the distribution observed in our retrograde mapping experiment, we found that *Nts, Tac2*, and *Crh* are broadly distributed across the rostral-to-caudal extent of the structure, with *Nts* and *Tac2* showing higher expression in the caudal aspect, and *Crh* displaying a more uniform distribution (Figures 2D–M). Consistent with our retrograde mapping, we observed significant differences in the anatomical distributions of the neuropeptides (Figures 2N,O; Supplementary Figure 2C), particularly within the more anterior regions of the BNST (Figure 2N). Within both the rostral and caudal BNST, *Nts-, Tac2-*, and *Crh*-expressing cells were predominantly non-overlapping; however, cells expressing some combination of two of three markers were more prevalent than those that expressed all three (Figures 2D–L). The largest amount of overlap of all three neuropeptide-encoding genes was located in the posterior oval region; however, this still represented only a small fraction of the total number of cells (Figures 2J,K).

To establish whether *Nts-, Tac2-*, and *Crh*-expressing cells within the BNST co-release amino acid neurotransmitters, we probed for the vesicular glutamate transporter 2 gene, *Slc17a6*, and the vesicular GABA transporter gene, *Scl32a1*, and in a separate experiment probed for the vesicular glutamate transporter 3 gene, *Slc17a8*, along with *Slc32a1* (Supplementary Figure 3). In both experiments, most peptide-expressing cells (>80%) co-labeled with *Slc32a1*. Few peptide-expressing cells (<3%) co-labeled with *Slc17a6* or *Slc17a8* alone, but we did observe populations of cells for all three peptides that co-expressed *Slc32a1* along with one of the glutamatergic markers (Supplementary Figure 3). The most prominent of these were *Nts*-expressing neurons and *Tac2*-expressing neurons that co-labeled with *Slc17a8* and *Slc32a1*. We also observed a number of cells that did not express any of the glutamatergic or GABAergic markers (Supplementary Figure 3).

**Figure 3:**
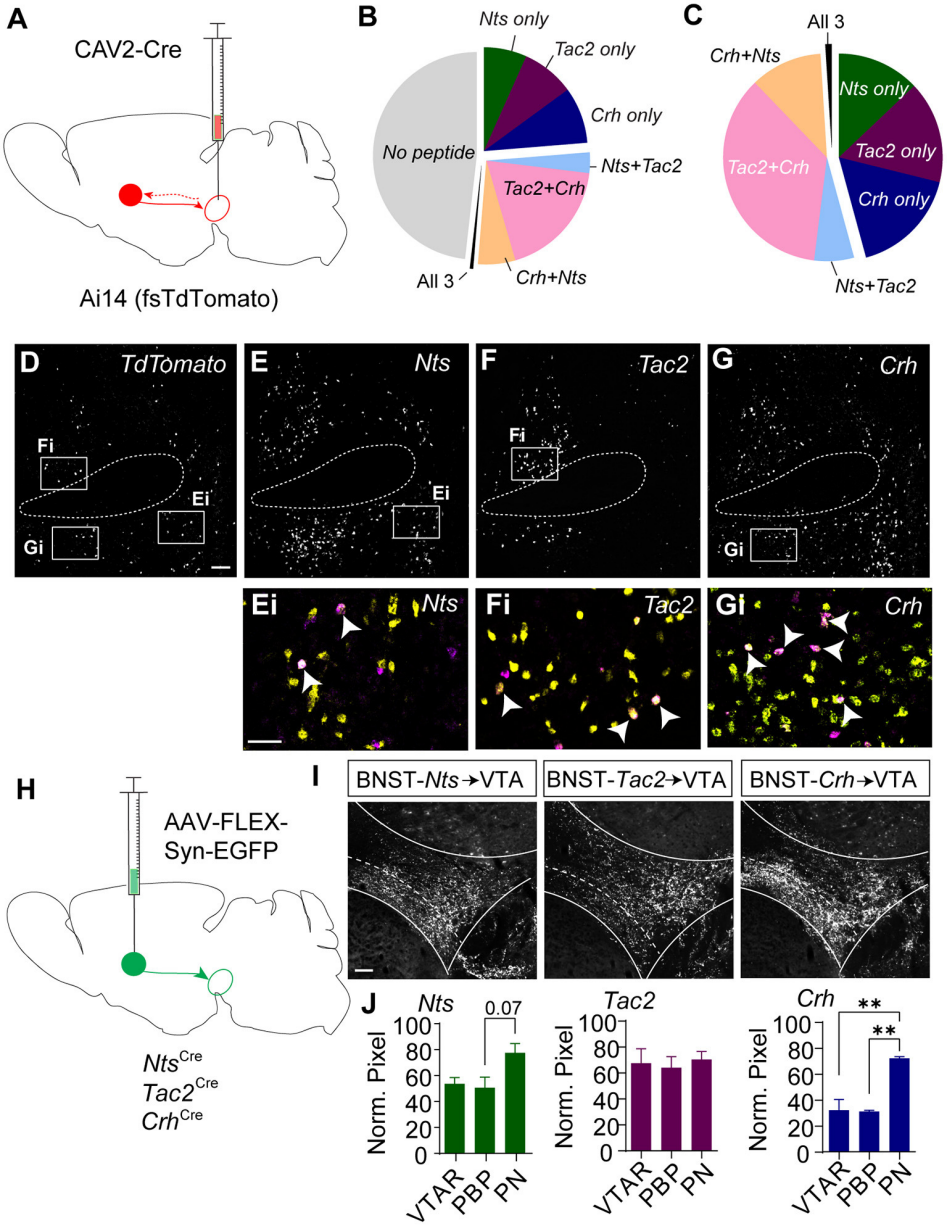
Retrograde and anterograde labeling of BNST to VTA *Nts, Tac2*, and *Crh* projections. **(A)** Schematic of CAV2-Cre injection into the VTA of an Ai14 (floxed-stop TdTomato) mouse to label VTA-projecting neurons. *In situ* hybridization was performed on BNST sections to probe for *TdTomato, Crh, Tac2*, and *Nts*. **(B)** Pie chart showing peptide expression in VTA-projecting TdTomato-positive neurons in the BNST (*n* = 776 total neurons from *N* = 7 mice). **(C)** Peptide distribution in VTA-projecting BNST neurons that expressed at least one peptide (*n* = 402 neurons from *N* = 7 mice). **(D–G)** Example image showing *in situ* for TdTomato **(D)**, *Nts* **(E)**, *Tac2* **(F)**, and *Crh* **(G)**. Scale bar = 100 μm. Ei-Gi are zoomed in views showing overlap of peptide markers (yellow) and TdTomato (magenta) and correspond to boxed regions in **(E–G)**. and indicated peptide (yellow). Scale bar = 50 μm. All images are of the same brain section, separated by peptide for ease of viewing. White arrows identify TdTomato-positive neurons labeled with a single peptide. **(H)** Schematic of injection of AAV-FLEX-synaptophysin-EGFP for BNST to VTA terminal mapping. **(I)** Example images of syn-EGFP labeling in the VTA in each Cre line. Scale bar = 100 μm. **(J)** Normalized pixel intensity in each VTA subregion [VTAR = rostral VTA, PBP = parabrachial pigmented nucleus, PN= paranigral VTA; *N* = 3 mice/group, One-way ANOVA Nts: *F*_(2, 6)_ = 4.522, *P* = 0.0634, Tukey's multiple comparisons PBP vs. PN P = 0.0747, Crh: *F*_(2, 6)_ = 22.69, *P* =0.0016, Tukey's multiple comparisons **P < 0.01]. Data in bar graphs are presented as mean ± SEM.

To resolve the degree of overlapping expression of *Nts, Tac2*, and *Crh* specifically in BNST projections to the VTA we injected the Ai14 reporter mouse line with CAV2-Cre in the VTA (Figure 3A) and performed RNAscope *in situ* hybridization for the three peptide genes along with a probe for the fluorescent reporter *tdTomato* (Figures 3B–G). Just over 50% of retrogradely labeled cells in the BNST expressed at least one of the three neuropeptide markers (Figure 3B). Of the tdTomato/neuropeptide co-labeled cells ~45% expressed only one marker (Figure 3C). Most cells that co-labeled expressed varying degrees of overlap in the combination of two of the neuropeptides, but very few cells were identified that co-labeled with all three markers (Figures 3B,C).

Based on the partially overlapping expression of the three neuropeptide markers, we asked whether BNST-*Nts*, BNST-*Tac2*, and BNST-*Crh* neurons have similar innervation patterns of the VTA. To achieve this, we injected *Nts-*Cre, *Tac2*-Cre, and *Crh*-Cre mice into the BNST with AAV1-FLEX-synaptophysin-EGFP (Figure 3H) and quantified the relative density of the projections into the subdivisions of the VTA. BNST-*Nts* inputs showed a partial, but non-significant bias toward innervation of the paranigral (PN) region (Figures 3I,J). BNST-*Tac2* inputs showed a nearly equal distribution of inputs to the rostral VTA (VTAR), parabrachial pigmented region (PBP), and the PN (Figures 3I,J). BNST-*Crh* inputs showed a significant bias toward innervation of the PN compared to either the PBP or VTAR (Figures 3I,J). We also examined projections from the BNST to the substantia nigra pars compacta (SNc), a dopaminergic region lateral to the VTA. BNST-*Nts* inputs were not different between the VTA and SNc (Supplementary Figure 4A). In contrast, BNST-*Tac2* inputs appeared denser in the SNc than in the VTA, which approached significance (Supplementary Figure 4B), and BNST-*Crh* inputs were significantly higher in the VTA, particularly to the PN subdivision (Supplementary Figure 4C).

### Similar and Distinct Encoding of Appetitive Information by BNST Inputs to the VTA

**Figure 4:**
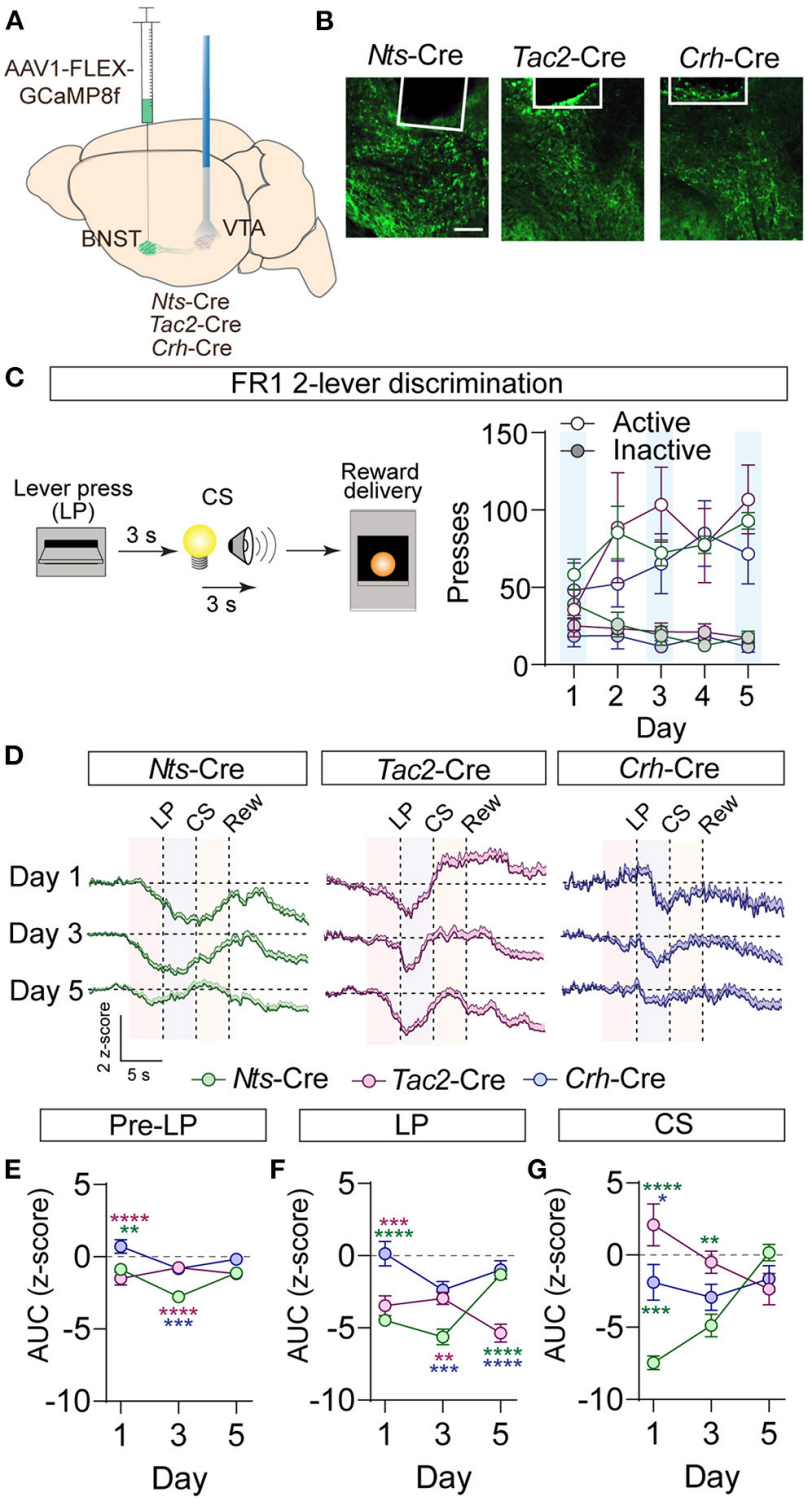
GCaMP imaging of BNST → VTA axon terminals during operant conditioning in *Nts-, Tac2-*, and *Crh*-Cre mice. **(A)** Schematic of AAV1-FLEX-GCaMP8f injection in the BNST of peptide Cre lines and fiber optic implant for photometry in the VTA. **(B)** Example images of fiber placement and GCaMP terminal expression. Scale bar = 100 μm. **(C)** Schematic of appetitive operant paradigm: a press on the active lever resulted in a 3 s delay followed by a 3 s cue delivery (tone plus light) that terminated with reward delivery. Right, number of presses on the active and inactive levers over 5 days of training (*n* = 7 *Nts*-Cre, 5 *Tac2*-Cre, and 4 *Crh*-Cre mice). **(D)** Average z-score (+SEM) of the ΔF/F of the GCaMP signal aligned to active lever press for all trials from all mice for days 1, 3, and 5 of training. **(E,F)** Area under the curve of the z-score for the 3 second period prior to the lever press **(E)**, following the lever press **(F)**, or during CS delivery **(G)**. [*n* = 7 *Nts*-Cre, 5 *Tac2*-Cre, and 4 *Crh*-Cre mice, 2-way RM ANOVA Pre-LP Interaction *F*_(4, 39)_ = 6.382, *P* = 0.0005, LP Interaction *F*_(4, 39)_ = 12.98, *P* < 0.0001, CS Interaction *F*_(4, 39)_ = 13.10, *P* < 0.0001, Tukey's multiple comparisons *P < 0.05, **P < 0.01, ***P < 0.001, ****P < 0.0001]. Data are presented as mean ± SEM.

Activation of GABAergic inputs to the VTA from the BNST is sufficient to drive appetitive behavioral responses (Jennings et al., 2013). To determine whether BNST-*Nts*, BNST-*Tac2*, or BNST-*Crh* inputs to the VTA respond to appetitive stimuli, we measured terminal calcium signals of these inputs to the VTA using the calcium sensor GCaMP and fiber photometry. *Nts-*Cre, *Tac2*-Cre, and *Crh*-Cre mice were injected with AAV1-FLEX-jGCaMP8f (Zhang et al., 2021) into the BNST and an optic imaging fiber was inserted over the VTA (Figures 4A,B; Supplementary Figure 5). GCaMP signals were measured during an instrumental cued two-lever discrimination task (Figure 4C) on the 1st, 3rd, and 5th days of conditioning. A single press on the active lever led to a 3 s delay followed by a 3 s compound cue (tone + light) that terminated with sucrose pellet delivery. There was no difference in behavioral performance on the task (active and inactive lever presses) between the three different Cre-driver lines (Figure 4C).

During conditioning, we observed a decrease in the GCaMP fluorescence just prior to the lever press in the BNST-*Nts* and BNST-*Tac2* inputs that persisted through the lever press period in both inputs, and through the CS period in the BNST-*Nts* inputs. Inhibition prior to the lever press was more prominent in the BNST-*Nts* inputs and diminished over the course of the conditioning days (Figures 4D–F; Supplementary Figures 6A–C). In contrast, the reduction in GCaMP fluorescence in the BNST-*Tac2* inputs during the lever press period was stronger on the final day of conditioning (Figure 4F; Supplementary Figures 6A–C). During the CS period, we observed an increase in fluorescence in the BNST-*Tac2* inputs that diminished over the course of the conditioning days (Figure 4G; Supplementary Figures 6A–C). BNST-*Crh* inputs to the VTA were only modestly responsive during these periods (Figures 4D–G; Supplementary Figures 6A–C).

**Figure 5:**
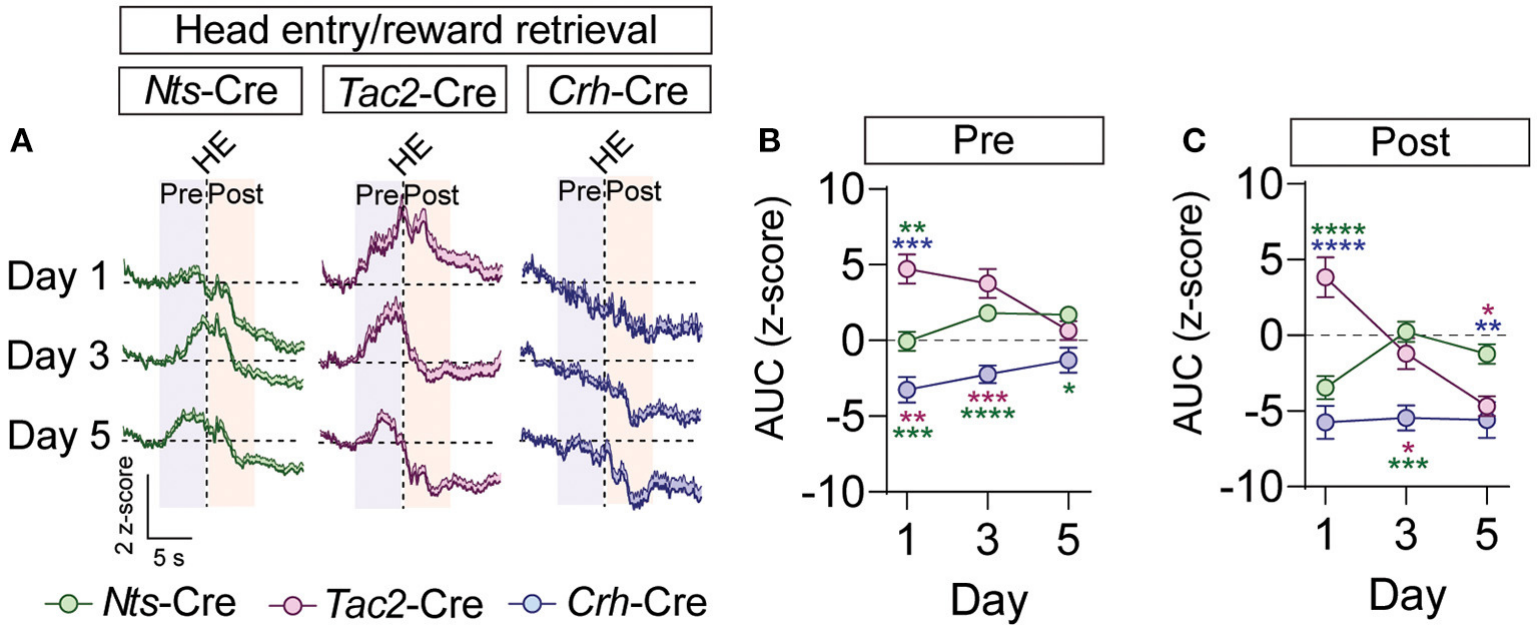
GCaMP imaging of BNST → VTA axon terminals during reward retrieval in *Nts-, Tac2-*, and *Crh*-Cre mice. **(A)** Average z-score (+SEM) of the ΔF/F of the GCaMP signal aligned to the first head entry into the food hopper following reward delivery for all trials from all mice for days 1, 3, and 5 of training. **(B,C)** Area under the curve of the z-score for the 5 second period prior to **(B)** or following **(C)** the head entry [*n* = 7 *Nts*-Cre, 5 *Tac2*-Cre, and 4 *Crh*-Cre mice, 2-way RM ANOVA Pre-HE Interaction *F*_(4, 39)_ = 5.825, *P* = 0.0009, Pre-HE Interaction *F*_(4, 39)_ = 11.77, *P* < 0.0001, Tukey's multiple comparisons *P < 0.05, **P < 0.01, ***P < 0.001, ****P < 0.0001]. Data are presented as mean ± SEM.

To determine whether these inputs respond to the reward, we analyzed GCaMP fluorescence during the head entry into the food port immediately following reward delivery. In the BNST-*Nts* inputs we observed a prominent decrease in fluorescence immediately following head entry that diminished across the days of conditioning (Figures 5A–C; Supplementary Figures 6D–F) and was preceded by an increase in fluorescence on the 3rd and 5th days of conditioning (Figures 5A–C; Supplementary Figures 6D–F). The BNST-*Tac2* inputs displayed a marked increase in fluorescence prior to the head entry that persisted immediately following the head entry (Figures 5A–C; Supplementary Figures 6D–F). This increase diminished over time, and we observed a prominent reduction in the fluorescence following head entry on the final day (Figures 5A,C; Supplementary Figures 6D–F). BNST-*Crh* inputs displayed a reduction in fluorescence that preceded head entry to the food port and persisted after the head entry, and this response was relatively stable across conditioning days (Figures 5A–C; Supplementary Figures 6D–F).

### Similar and Distinct Encoding of Aversive Information by BNST Inputs to the VTA

**Figure 6:**
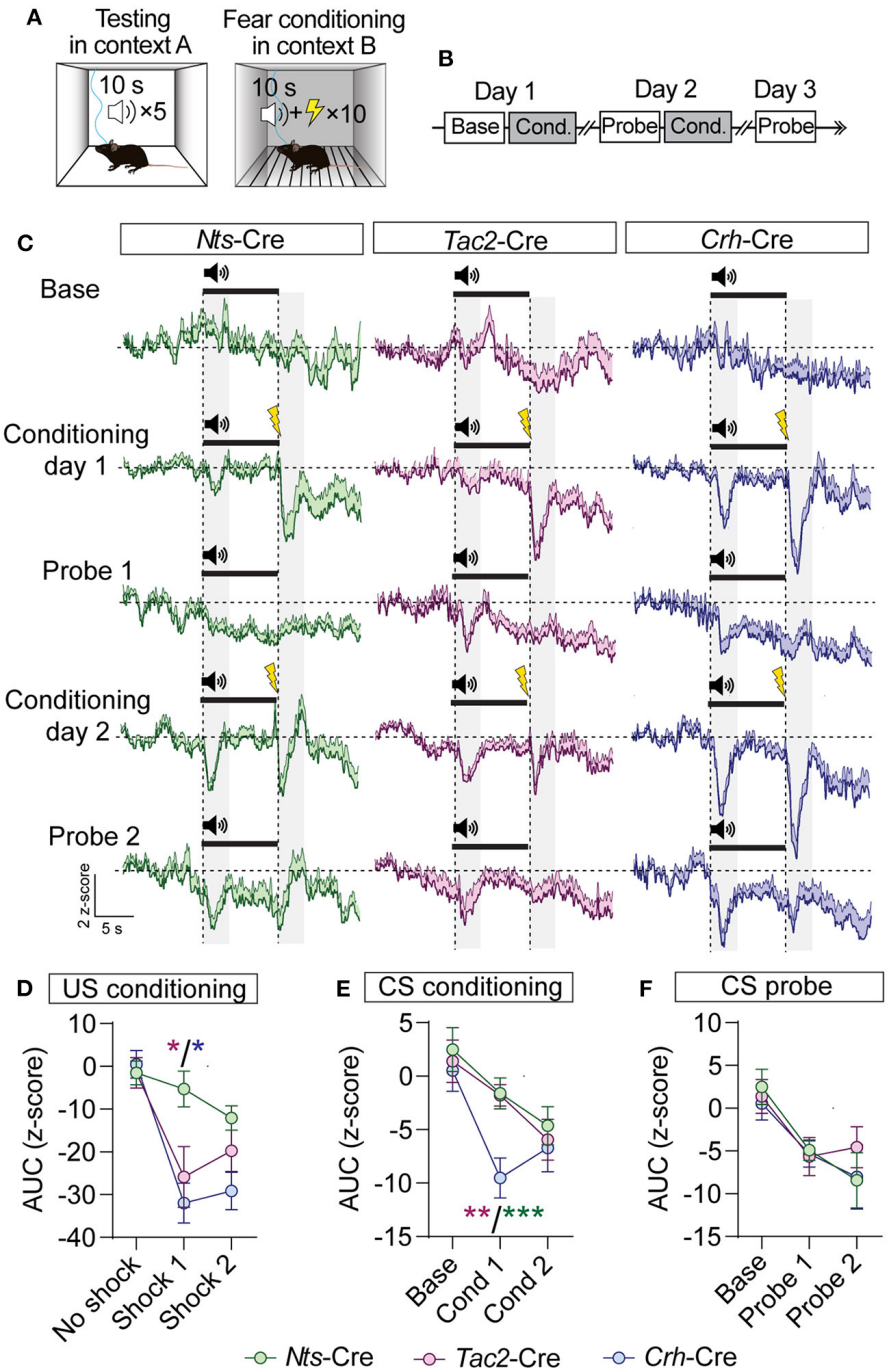
GCaMP imaging of BNST → VTA axon terminals during fear conditioning in *Nts-, Tac2-*, and *Crh*-Cre mice. **(A,B)** Schematic of fear conditioning paradigm. Mice received baseline and probe sessions (cue delivery only) in context A, and conditioning sessions (cue + shock) in context B. **(C)** Average z-score (+SEM) of the ΔF/F of the GCaMP signal aligned to the cue onset for all trials from all mice. **(D–F)** Area under the curve of the z-score for the 3 s period following shock delivery **(D)**, following the onset of the CS during conditioning sessions **(E)**, or following the onset of the CS during probe sessions **(F)** [*n* = 5 *Nts*-Cre, 6 *Tac2*-Cre, and 5 *Crh*-Cre mice, 2-way RM ANOVA US Conditioning Interaction *F*_(4, 26)_ = 3.637, *P* = 0.0175, CS Conditioning Effect of Cre line *F*_(2, 13)_ = 4.716, *P* = 0.0288, Effect of Session *F*_(1.3, 16.89)_ = 11.90, *P* = 0.0017, CS Probe Effect of Session *F*_(1.624, 21.12)_ = 11.87, *P* = 0.0007, Tukey's multiple comparisons *P < 0.05, **P < 0.01, ***P < 0.001]. Data are presented as mean ± SEM.

GABAergic BNST inputs to the VTA are inhibited by an aversive footshock (Jennings et al., 2013). To determine whether neuropeptidergic inputs we identified from the BNST to the VTA are similarly inhibited by aversive stimuli we monitored GCaMP fluorescence in the VTA of *Nts-*Cre, *Tac2*-Cre, and *Crh*-Cre mice injected with AAV1-FLEX-jGCaMP8f into the BNST, as above. Mice were conditioned in a classical fear conditioning paradigm in which a tone (10 s) was presented in a neutral context A, followed by pairing the tone (CS) with delivery of a foot shock (US, 0.3 mA, 0.5 s) in context B. Mice were tested for 3 days in the neutral context (day 1 baseline) and conditioned (10-CS/US pairings) on days one and two (Figures 6A,B).

During baseline we did not observe significant changes in fluorescence during CS presentation (Figure 6C). During conditioning we observed time locked reductions in GCaMP fluorescence following both the CS and US presentations in all three groups of mice (Figures 6C–E). During the probe trials on days 2 and 3, we also observed time locked reductions in GCaMP fluorescence following CS presentation (Figures 6C,F). We observed similar time locked responses to the CS and US in BNST-*Nts*, BNST-*Tac2*, or BNST-*Crh* inputs to the VTA; however, US responses were significantly weaker in BNST-*Nts* inputs on day 1 of conditioning (Figures 6D,E). BNST-*Crh* inputs showed the strongest US responses on both conditioning days, though they were not significantly different than BNST-*Tac2* inputs. Responses in BNST-*Crh* inputs were significantly stronger to the CS during the first day of conditioning compared to either BNST-*Nts* or BNST-*Tac2* (Figure 6E).

## Discussion

Utilizing a Cre-dependent retrograde viral mapping strategy previously validated to establish neurotransmitter- and neuropeptide-specific inputs to a brain region of interest (Sanford et al., 2017; Ahmadlou et al., 2018; Kohl et al., 2018; Soden et al., 2020; Fellinger et al., 2021), we found that NTS, NkB, and CRF inputs to the VTA arise from numerous anatomical locations. Although tropism for specific viruses in the brain can influence the degree of retrograde mapping, we found a distribution of NTS inputs to the VTA similar to what had been described using an alternative strategy where a retrograde tracer was injected into the VTA of mice expressing GFP in *Nts* neurons (Woodworth et al., 2018a). To our knowledge a similar brain-wide analysis of NkB and CRF inputs to the VTA has not been previously reported.

Of the numerous NTS, NkB, and CRF inputs to the VTA, we find that the BNST is a major source of all three neuropeptides, with all three showing similar numbers of neurons projecting to the VTA. Analysis of another extended amygdala region, the lateral subdivision of the central nucleus of the amygdala (CeAL), found that more than half of the neurons express all three neuropeptide genes, *Nts, Crh, and Tac2* and all are GABAergic (Kim et al., 2017). Similar to the CeAL, we find that the majority of BNST *Nts, Crh, and Tac2* expressing neurons are GABAergic; however, very few express all three neuropeptides. These results are consistent with single cell sequencing of the BNST that identified *Nts* and *Crh* expressing neurons as largely distinct (Rodriguez-Romaguera et al., 2020; Ortiz-Juza et al., 2021). Here, we find that ~12% of BNST neurons express both *Crh* and *Nts*, indicating that they are predominantly non-overlapping populations.

In the BNST projections to the VTA, we find that just over half of the neurons express some combination of two of the three neuropeptides, but again very few expressed all three. We also observed that nearly half of all the inputs to the VTA from the BNST do not express any of the three neuropeptides investigated here, suggesting that these neurons are likely to release other neuropeptides into the VTA, such as the endogenous opioids enkephalin, dynorphin, and nociceptin that are known to modulate neural activity in this region (Kash et al., 2015; Giardino and Pomrenze, 2021). Indeed, we previously identified a dynorphin-expressing BNST to VTA population that contributed to anxiety-like behavior, but not conditioned threat discrimination (Fellinger et al., 2021). In this study, we observed that stimulation of BNST-*Pdyn* neurons was anxiogenic, similar to what was reported for stimulation of BNST-glutamate neurons (Jennings et al., 2013). In contrast, stimulation of GABA neurons in the BNST is anxiolytic (Jennings et al., 2013). Based on the high degree of overlapping expression of *Nts, Crh*, and *Tac2* with Vgat in the BNST, we hypothesize that these peptidergic inputs would also be anxiolytic and largely non-overlapping the BNST-*Pdyn* inputs.

Although there is not yet a clear resolution of how inhibitory GABA neurons that co-release stimulatory peptides function at the neurocircuit level, there is abundant evidence for the co-release of neurotransmitters and neuropeptides with ostensibly opposing actions in the central nervous system (Nusbaum et al., 2017). Because the majority of GABAergic inputs to the VTA from the BNST inhibit VTA GABA neurons to disinhibit dopamine neurons (Jennings et al., 2013; Soden et al., 2020), this action could work coordinately with direct peptide excitation of dopamine neurons, which are enriched for the expression of many peptide receptors (Chung et al., 2017). Our studies of calcium dynamics in BNST to VTA terminals cannot determine what role individual peptides or fast transmitters are playing in altering downstream neuronal activity, but they do identify specific behavioral epochs during which these inputs may be influencing VTA activity.

NTS is broadly implicated in the regulation of feeding (Ramirez-Virella and Leinninger, 2021), whereas NkB and CRF have been shown to regulate arousal, stress, and anxiety (Dedic et al., 2018; Zelikowsky et al., 2018). During instrumental conditioning, we found that BNST-*Nts* and BNST-*Tac2* projections to the VTA were largely inhibited during the lever press and cue period, but BNST-*Crh* inputs were largely unresponsive. In contrast, during reward retrieval both BNST-*Nts* and BNST-*Tac2* projections were activated and BNST-*Crh* inputs were inhibited. Activation of BNST-*Nts* and BNST-*Tac2* projections to the reward is consistent with appetitive and arousing nature of the reward, but why these inputs are suppressed during the performance of the instrumental action is not clear. One possibility is that decreased activity of these GABAergic inputs relieves direct inhibition of VTA GABA neurons, allowing them to suppress extraneous activity during the instrumental actions. This is consistent with previous observations that only a subset of dopamine neurons are activated by cues and kinematics during a cued maze task (Engelhard et al., 2019). In contrast, the majority of dopamine neurons are activated by a reward (Romo and Schultz, 1990), so activation of BNST-*Nts* and BNST-*Tac2* inputs during reward retrieval would promote the disinhibition of dopamine neurons, as well as direct excitation via peptide activity, consistent with the reinforcing effects of stimulating BNST-GABA inputs to the VTA (Jennings et al., 2013).

It is also interesting to note that although we see some overlapping expression of the neuropeptide projections to the VTA, the patterns of terminal labeling and the response profiles during the instrumental behavior are not uniform. We speculate that these differences may originate from the cells with non-overlapping expression, but more sophisticated intersectional strategies will be required to further resolve this question. It is also possible that the degree of axon branching and the number of terminals per neuron may vary between different BNST cell types, which could also contribute to the differences we observe between Cre lines.

In response to aversive stimuli, we observed time locked inhibitions of all three inputs to the VTA, consistent with previous reports of inhibition of BNST-GABA inputs in response to foot shock (Jennings et al., 2013). Notably, none of the inputs responded to the presentation of the CS tone prior to conditioning, but all three showed an inhibition to CS onset in a neutral context following 1 or 2 days of conditioning, reflecting a learned association. BNST-*Crh* inputs were the most responsive to the US and CS during conditioning, with BNST-*Nts* inputs being the least responsive. It is possible that this is a reflection of the partial overlapping expression of *Nts* and *Crh* and *Tac2* and *Crh*. If the majority of BNST-*Crh* inputs are inhibited by the aversive stimuli and only a subset of *Nts* neurons are inhibited, it is likely that the BNST-*Nts* inputs that are suppressed are those with overlapping *Crh* expression.

Previous calcium imaging experiments in BNST-*Crh* neurons demonstrated that these cells respond to painful stimuli (Yu et al., 2021) and restraint stress (Luchsinger et al., 2021). In response to an acute noxious stimulus BNST-*Crh* neurons responded with approximately equal numbers having increased or decreased calcium signals (Yu et al., 2021). Based on these observations, it is likely that a portion of the BNST-*Crh* neurons that were inhibited by a pain-inducing stimulus are VTA projection neurons. Consistent with the inhibition of a large number of VTA dopamine neurons by aversive stimuli (Brischoux et al., 2009), suppression of BNST-GABA inputs expressing *Crh, Tac2*, or *Nts* would disinhibit VTA GABA neurons allowing for the inhibition of dopamine producing cells in the region. In addition to previous evidence that BNST-GABA neurons predominantly synapse onto VTA-GABA neurons, analysis of BNST-CRF inputs to the VTA revealed that CRF-positive terminals predominantly localize around the cell bodies of non-dopamine producing cells in the VTA (Dabrowska et al., 2016). Thus, it is likely that BNST-*Crh* neurons form disinhibitory circuits within the VTA.

In both the instrumental and fear conditioning calcium imaging data we observed considerable plasticity across the conditioning days. This is consistent with dynamic changes in dopamine neuron activity that have been reported in these types of assays (Volman et al., 2013). The plasticity observed during fear conditioning was fairly uniform between groups and appeared to reflect the development of a conditioned response to the shock-predicting cue. The dynamic changes observed across days of appetitive conditioning were more complex and variable, involving shifts in the timing of the calcium response onset and the magnitude of the response, which may reflect changes in expectation regarding the outcome of a given action or cue as animals become well trained.

In summary, there are numerous sources of NTS, NkB, and CRF to the VTA. The diversity of these inputs likely contributes to the diversity of functions regulated by the mesolimbic system. In addition to highlighting the range of sources of these neuropeptides, we find that even when derived from the same anatomical location these VTA input neurons can have distinct expression of several neuropeptides. Consistent with the shared and distinct molecular identity of BNST inputs, we observed both common and unique responses to appetitive and aversive stimuli. In combination with the partially overlapping expression of neuropeptide receptors in the VTA, this organization would allow these neuropeptides to modulate VTA activity either independently or coordinately depending on the specificity of the behavior.

## Materials and Methods

### Mice

All procedures were approved and conducted in accordance with the guidelines of the Institutional Animal Care and Use Committee of the University of Washington. Mice were group-housed on a 12-h light/dark cycle with ad libitum food and water. Approximately equal numbers of male and female mice were used for all experiments. Crh-Cre (Stock number 012704), Nts-Cre (Stock number 017525), *Tac2*-Cre (Stock number 021878), and Ai14 TdTmto (Stock number 007914) mice are available from Jackson Laboratories.

### Viruses

AAV1 and CAV viruses were produced in-house as described (Gore et al., 2013).

### Surgery

Mice were anesthetized with isoflurane before and during viral injection. Mice were injected at ~6–8 weeks of age, and experiments were performed 2–4 weeks following injection. VTA coordinates were M-L: ±0.5, A-P: −3.25, D-V: −4.25. BNST coordinates were M-L: ±0.8, A-P: +0.4, D-V: 4.3. Values are in mm, relative to bregma. For the VTA A-P values were adjusted for bregma-lambda distance using a correction factor of 4.21 mm. For Z values the syringe was lowered 0.5 mm past the indicated depth and raised up at the start of the injection. Injection volume was 500 nl. Fiber optic cannulas for photometry (400 μm fiber, 0.66 NA, 1.25 mm ferrule) were from Doric. The fibers were implanted in the VTA at a depth of −4.1 mm from the skull at an angle of 5°.

### Retrograde Input Mapping

At least 3 weeks following injection of 500 nl CAV2-FLEX-zsGreen virus into the VTA, mice were euthanized and perfused with 4% paraformaldehyde. 30 μm frozen brain sections were collected and mounted on glass slides. One brain section per approximately every 120 μm was imaged at 10 × magnification using a Keyence BZ-X710 fluorescent microscope and cells in each brain region were counted by an experienced investigator. Brain regions were included if we observed retrograde labeling in at least half of injected animals.

### Synaptophysin-GFP Input Mapping

At least 2 weeks following injection of AAV1-FLEX-synaptophysinGFP, mice were euthanized and perfused with 4% paraformaldehyde. 30 μm frozen brain sections were collected, and 8 sections spanning the rostral-caudal extent of the VTA were selected and stained overnight with a Rabbit anti GFP antibody (Invitrogen A11122, 1:2000). Images were collected at 10× magnification using a Keyence BZ-X710 fluorescent microscope and analyzed using ImageJ software. Images were background subtracted and mean pixel intensity and integrated pixel density were measured for each subregion. For normalized intensity plots all subregions from an individual animal were normalized to the highest intensity VTA subregion from that animal.

### *In situ* Hybridization

RNAscope (Advanced Cell Diagnostics) *in situ* hybridization was performed according to manufacturer's instructions on 20 μm fresh-frozen coronal brain sections. Version 1 was used for peptide expression and *Slc32a1*/*Slc17a6* expression in the BNST. Version 2 was used for peptide receptor expression in the VTA. 4-channel Version 2 was used for retrograde CAV2 experiments. Images were collected using a Leica SP8 confocal microscope and were quantified using HALO software (Indica Labs), except for the retrograde experiment, which was quantified manually using ImageJ software. The rostral BNST was defined as +0.5 to +0.14 mm from bregma, and the caudal BNST from +0.02 to −0.22 mm from bregma.

### Behavior and Fiber Photometry

Mice were food restricted to 85% of ad libitum body weight. Mice received one 30-min session of acclimation to the photometry patch cord (Doric Lenses) in the operant chamber (MedAssociates), followed by two pre-training days in which the house light was illuminated and both levers were extended. A press on either lever led to extinction of the house light and immediate pellet delivery, followed by a 3 s ITI. Mice were allowed a maximum of 20 pellets per day in pre-training. Next, mice experienced 5 days of delayed cue training, in which both levers were extended but only one lever was active. A press on the active lever led to a 3 s delay, followed by a delivery of a 3 s compound cue (lever light plus 4 KHz tone), followed by pellet delivery. Each rewarded press was followed by a 12.5 s ITI. Training sessions lasted for 1 h.

Fear conditioning was performed in the same operant boxes used for operant conditioning. However, for the probe sessions the floor and walls of the box were covered with solid white plastic to alter the context. Mice received a probe session in the morning in which they received 5 CS presentations (10 s 10 KHz tone), and a training session in the afternoon in which the CS coterminated with a 0.5 s 0.3 mA footshock (10 trials). On day 3 the mice only received a probe trial.

The imaging patch cord was photobleached prior to all recording sessions. Recordings were made using an RZ5 BioAmp Processor and Synapse software (Tucker Davis Technologies). A 465 nm LED (531-Hz, sinusoidal, Doric Lenses) was used to excite GCaMP. LED intensity was measured at the tip of the optic fiber prior to each recording session and set to approximately 40 μW. GCaMP fluorescence (525 ± 25 nm) was returned through the same patch cord, bandpass filtered, and recorded by the RZ5. A 405 nm LED (211-Hz, sinusoidal) was used to monitor the isosbestic signal. The 531-Hz and 211-Hz signals were extracted by Synapse software at a sampling rate of 1,017.25 Hz. Lever press, head entry, CS, and US events were synchronized to the photometry recording by TTL signals. Custom Python code was used to downsample, extract and analyze the GCaMP signal surrounding each stimulus event. A 30 s window was extracted surrounding each stimulus (10 s prior and 20 s following) and the first 4 s of this window was used as a baseline to calculate Z-score of the ΔF/F. Area under the curve was calculated for the indicated time periods for each animal based on the average z-score across all trials for that animal for that session.

Proper targeting of viral injections and fiber placement were confirmed with *post-hoc* immunohistochemistry. Animals with missed viral injections or significant viral spread outside the targeted region were excluded from analyses.

### Statistics

All data were analyzed for statistical significance using Prism software (GraphPad Prism 9). The Geisser-Greenhouse correction was used to correct for unequal variability of differences in repeated-measures ANOVA tests. All behavioral and photometry assays were repeated in a minimum of two cohorts with similar replication of results.

## Data Availability Statement

The raw data supporting the conclusions of this article will be made available by the authors, without undue reservation.

## Ethics Statement

The animal study was reviewed and approved by University of Washington IACUC.

## Author Contributions

MS and LZ designed the experiments, analyzed the data, and wrote the manuscript. MS, JY, and BC performed the fiber photometry recordings and data analysis. MS and BC analyzed the RNAscope. AR, BC, and JY performed histology. JE developed the operant paradigm and assisted with photometry analysis. MS performed RNAscope and retrograde mapping. All authors contributed to the article and approved the submitted version.

## Funding

This work was supported by National Institutes of Health grant R01 DA044315 (LZ) and the Brain & Behavior Research Foundation NARSAD Young Investigator Award (MS). This work was also supported by the University of Washington Center of Excellence in Opioid Addiction Research (P30 DA048736) and the University of Washington W. M. Keck Microscopy Center (NIH grant S10 OD016240).

## Conflict of Interest

The authors declare that the research was conducted in the absence of any commercial or financial relationships that could be construed as a potential conflict of interest. The reviewer MP declared a past co-authorship with the author LZ to the handling editor.

## Publisher's Note

All claims expressed in this article are solely those of the authors and do not necessarily represent those of their affiliated organizations, or those of the publisher, the editors and the reviewers. Any product that may be evaluated in this article, or claim that may be made by its manufacturer, is not guaranteed or endorsed by the publisher.
