# Supplementary material for: Distinct Encoding of Reward and Aversion by Peptidergic BNST Inputs to the VTA
Source: Front Neural Circuits. 2022 Jul 4;16:918839. doi: 10.3389/fncir.2022.918839 (PMC9289195; doi:10.3389/fncir.2022.918839)
Supplement: Supplementary file 1 [file Data_Sheet_1.PDF]

Supplementary Information

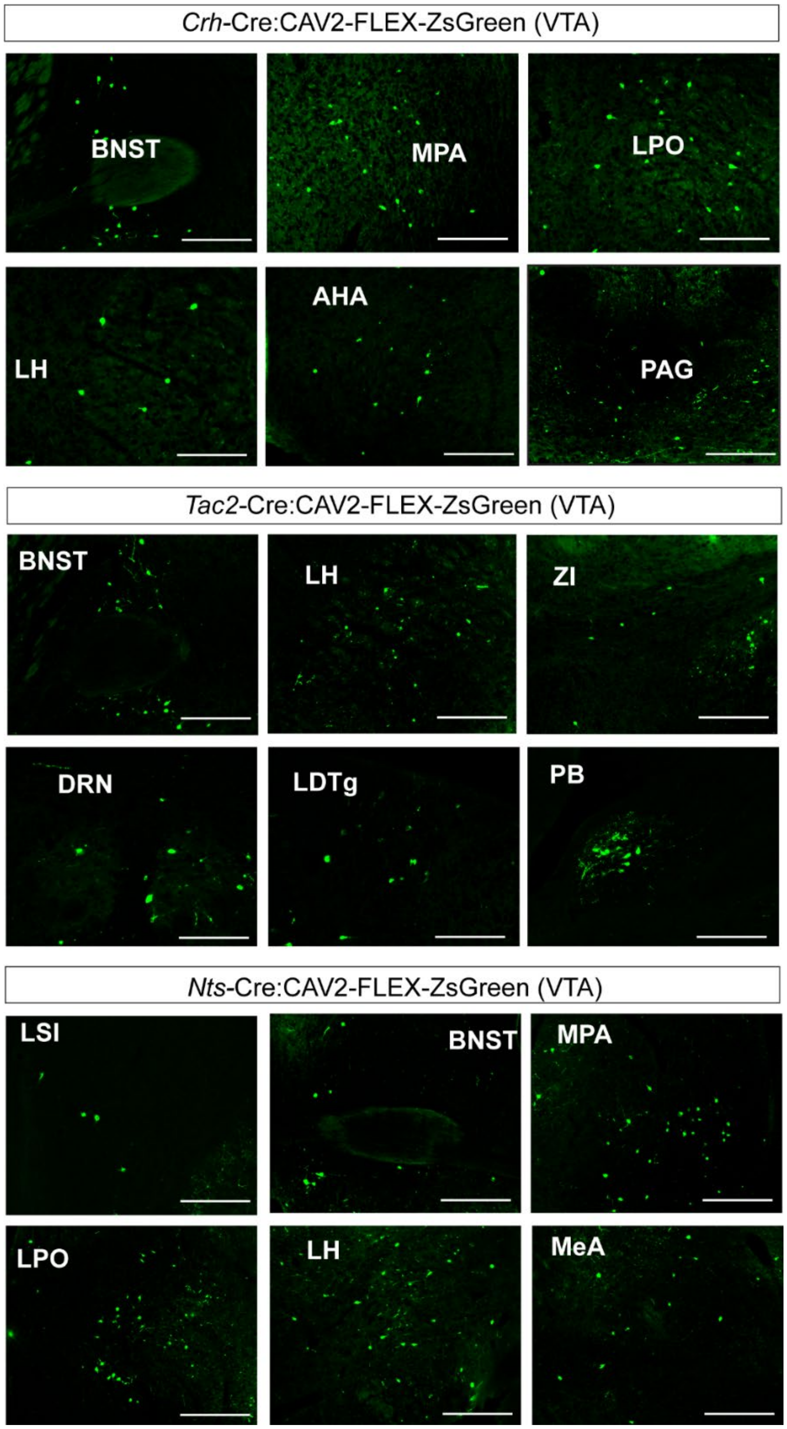

Supplemental Figure 1. Example images of zsGreen retrogradely labeled cells in various brain regions from each Cre line. Scale bars = 250 μm.

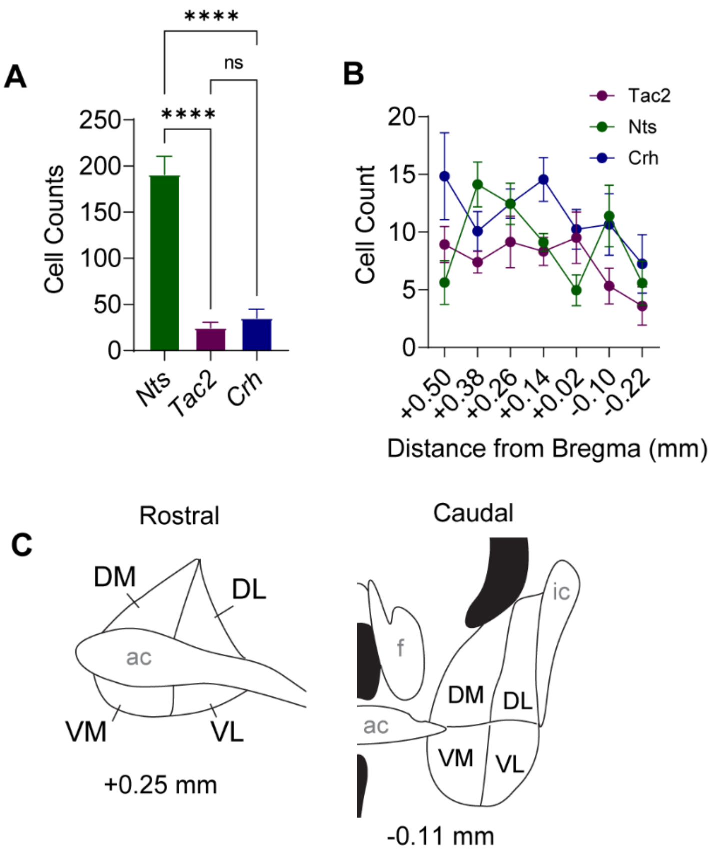

Supplemental Figure 2. (A) Counts of retrogradely labeled cells in the LH. **(B)** Rostral-caudal distribution of retrogradely labeled neurons in the BNST. **(C)** Designations of subregions in rostral and caudal BNST. DM: dorsomedial, DL: dorsolateral, VM: ventromedial, VL: ventrolateral, ac: anterior commissure, f: fornix, ic: internal capsule.

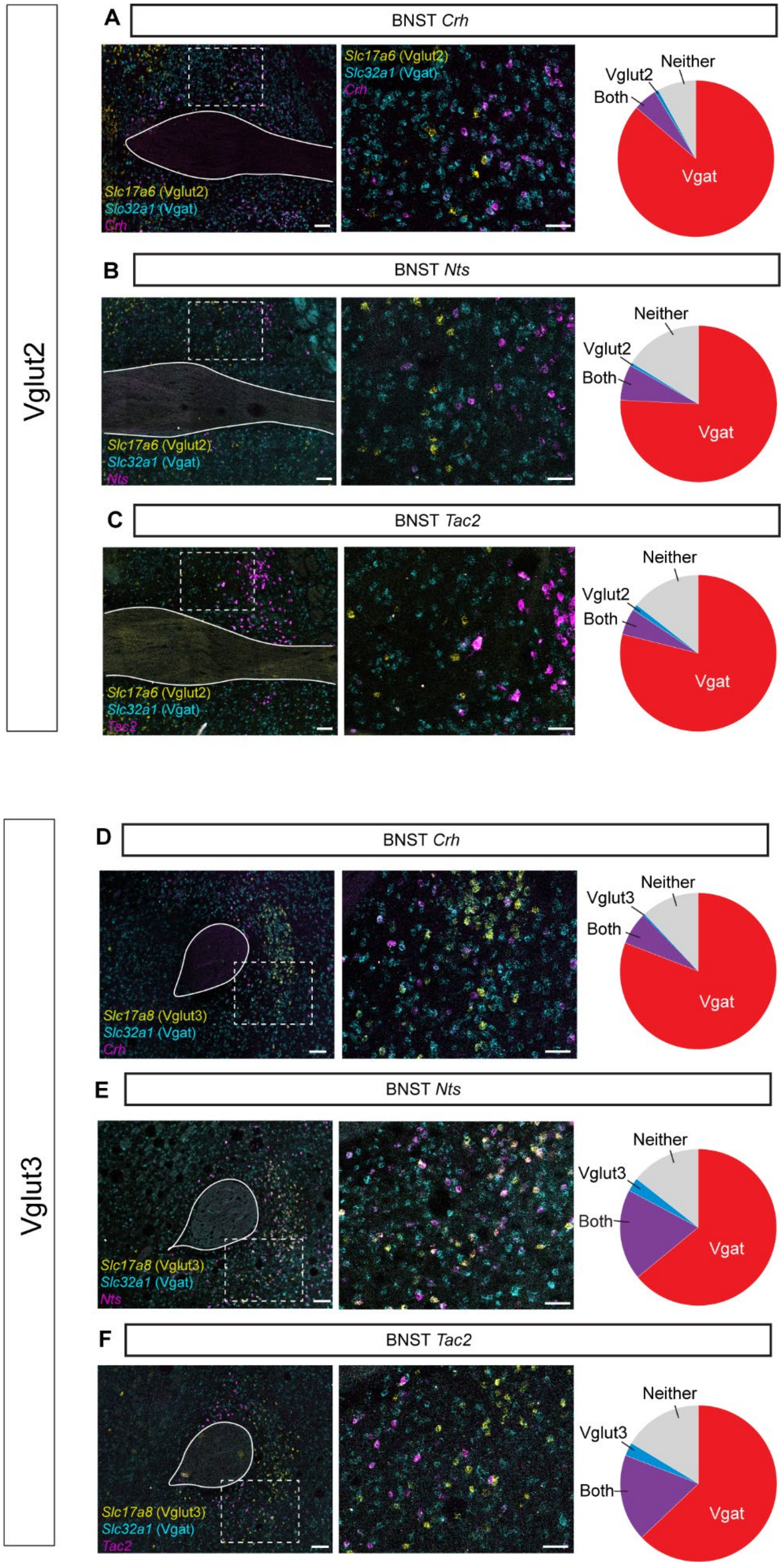

Vglut2
A
BNST *Crh*
Slc17a6 (Vglut2)
Slc32a1 (Vgat)
Crh
Neither
Vglut2
Both
Vgat
B
BNST *Nts*
Slc17a6 (Vglut2)
Slc32a1 (Vgat)
Nts
Neither
Vglut2
Both
Vgat
C
BNST *Tac2*
Slc17a6 (Vglut2)
Slc32a1 (Vgat)
Neither
Vglut2
Both
Vgat
Vglut3
D
BNST *Crh*
Slc17a8 (Vglut3)
Slc32a1 (Vgat)
Crh
Neither
Vglut3
Both
Vgat
E
BNST *Nts*
Slc17a8 (Vglut3)
Slc32a1 (Vgat)
Nts
Neither
Vglut3
Both
Vgat
F
BNST *Tac2*
Slc17a8 (Vglut3)
Slc32a1 (Vgat)
Tac2
Neither
Vglut3
Both
Vgat

Supplemental Figure 3. (A-C) Example images of *in situ* for *Slc17a6* (Vglut2), *Slc32a1* (Vgat), and *Crh* (A), *Nts* (B), or *Tac2* (C), and pie chart showing Vglut2 and Vgat expression in peptide-positive neurons (cells counted in n=3 sections from each of N=4 mice for each peptide). Left: scale bar = 100 µm. Right: scale bar = 50 µm. **(D-F)** Example images of *in situ* for *Slc17a8* (Vglut3), *Slc32a1* (Vgat), and *Crh* (D), *Nts* (E), or *Tac2* (F), and pie chart showing Vglut3 and Vgat expression in peptide-positive neurons (cells counted in n=3 sections from each of N=3 mice for each peptide). Left: scale bar = 100 µm. Right: scale bar = 50 µm.

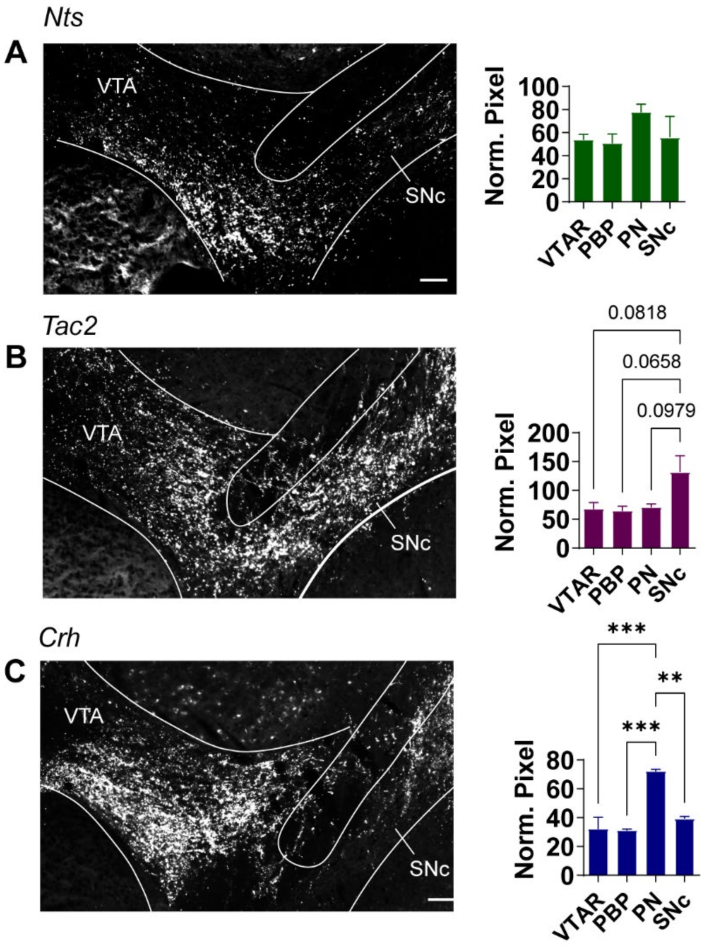

Supplemental Figure 4. (A-C) Left: Example images of syn-EGFP labeling in the VTA and SNc in each Cre line. Scale bar = 100 µm. Right: Quantification of normalized pixel intensity in each subregion (N=3 mice/group, One-way ANOVA Tac2: $F_{(3,8)}$=4.124, P=0.0484, Tukey's multiple comparisons as listed, Crh: $F_{(3,8)}$=20.17, P=0.0004, Tukey's multiple comparisons **P<0.01, ***P<0.001). Note: quantification of VTA subdivisions are the same as those presented in Figure 3. Data in bar graphs are presented as mean ± SEM.

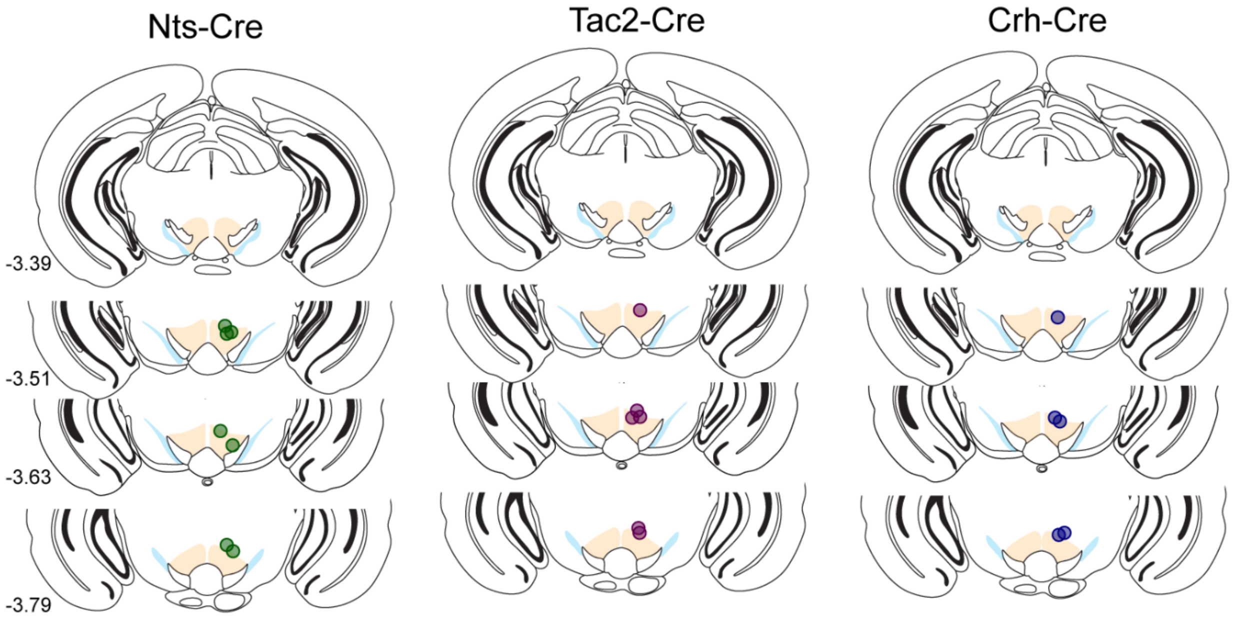

Supplemental Figure 5. Photometry fiber placements in the VTA. Orange shading represents the VTA, blue shading represents the SNc. Numbers are mm from bregma.

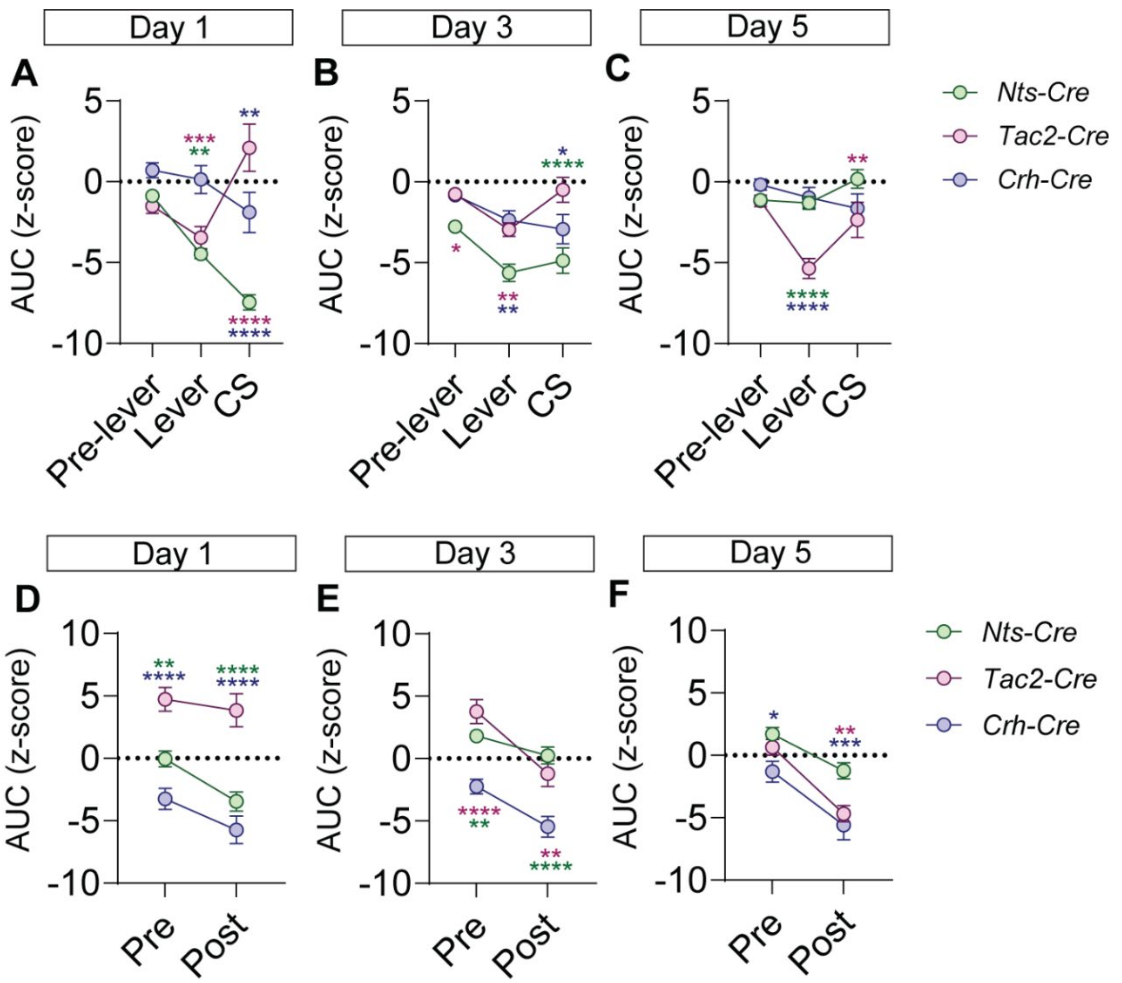

Supplemental Figure 6. (A-C) Area under the curve of the z-score for the 3 second period prior to the lever press, following the lever press, or during CS delivery on Day 1 (A), Day 3 (B), or Day 5 (C) of training (n=7 *Nts*-Cre, 5 *Tac2*-Cre, and 4 *Crh*-Cre mice, 2-way RM ANOVA Day 1 Interaction $F_{(4,39)}$=18.42, P<0.0001, Day 3 Effect of Cre Line $F_{(2,39)}$=24.24, P<0.0001, Effect of Time Period $F_{(2,39)}$=9.834, P=0.0003, Day 5 Interaction $F_{(4,39)}$=4.764, P=0.0032, Tukey's multiple comparisons *P<0.05, **P<0.01, ***P<0.001, ****P<0.0001). **(D-E)** Area under the curve of the z-score for the 5-second period before or after the head entry on Day 1 (D), Day 3 (E), or Day 5 (F) of training (n=7 *Nts*-Cre, 5 *Tac2*-Cre, and 4 *Crh*-Cre mice, 2-way RM ANOVA Day 1 Effect of Cre Line $F_{(2,26)}$=41.52, P<0.0001, Effect of Time Period $F_{(1,26)}$=8.542, P=0.0071, Day 3 Effect of Cre Line $F_{(2,26)}$=23.93, P<0.0001, Effect of Time Period $F_{(1,26)}$=25.72, P<0.0001, Day 5 Effect of Cre Line $F_{(2,26)}$=13.76, P<0.0001, Effect of Time Period $F_{(1,26)}$=48.93, P<0.0001, Tukey's multiple comparisons *P<0.05, **P<0.01, ***P<0.001, ****P<0.0001). Data are presented as mean ± SEM.
